# Defect Detection and 3D Reconstruction of Complex Urban Underground Pipeline Scenes for Sewer Robots

**DOI:** 10.3390/s24237557

**Published:** 2024-11-26

**Authors:** Ruihao Liu, Zhongxi Shao, Qiang Sun, Zhenzhong Yu

**Affiliations:** 1 School of Mechatronics Engineering, Harbin Institute of Technology, Harbin 150001, China; 2 Hefei Intelligent Robot Institute, Hefei 230601, China

**Keywords:** urban pipeline, defect detection, YOLO network, lightweight, edge deployment, 3D reconstruction

## Abstract

Detecting defects in complex urban sewer scenes is crucial for urban underground structure health monitoring. However, most image-based sewer defect detection models are complex, have high resource consumption, and fail to provide detailed damage information. To increase defect detection efficiency, visualize pipelines, and enable deployment on edge devices, this paper proposes a computer vision-based robotic defect detection framework for sewers. The framework encompasses positioning, defect detection, model deployment, 3D reconstruction, and the measurement of realistic pipelines. A lightweight Sewer-YOLO-Slim model is introduced, which reconstructs the YOLOv7-tiny network by adjusting its backbone, neck, and head. Channel pruning is applied to further reduce the model’s complexity. Additionally, a multiview reconstruction technique is employed to build a 3D model of the pipeline from images captured by the sewer robot, allowing for accurate measurements. The Sewer-YOLO-Slim model achieves reductions of 60.2%, 60.0%, and 65.9% in model size, parameters, and floating-point operations (FLOPs), respectively, while improving the mean average precision (mAP) by 1.5%, reaching 93.5%. Notably, the pruned model is only 4.9 MB in size. Comprehensive comparisons and analyses are conducted with 12 mainstream detection algorithms to validate the superiority of the proposed model. The model is deployed on edge devices with the aid of TensorRT for acceleration, and the detection speed reaches 15.3 ms per image. For a real section of the pipeline, the maximum measurement error of the 3D reconstruction model is 0.57 m. These results indicate that the proposed sewer inspection framework is effective, with the detection model exhibiting advanced performance in terms of accuracy, low computational demand, and real-time capability. The 3D modeling approach offers valuable insights for underground pipeline data visualization and defect measurement.

## 1. Introduction

Sewers constitute critical infrastructure in urban areas, and their effective operation is paramount for safeguarding urban facilities, personnel, and property [1]. The timely detection and repair of pipe defects are crucial for mitigating extensive damage and minimizing repair costs. Unfortunately, the concealed nature of underground drainage pipelines allows internal defects to accumulate unnoticed until an accident occurs [2]. Hence, regular inspections of pipelines during both the construction and usage stages are indispensable, facilitating the early identification and repair of any defects that may arise. Pipeline inspection technologies encompass acoustic inspection, electromagnetic inspection, and visual inspection. Among these, manual inspection using closed-circuit television (CCTV) remains the predominant technique [3,4]. However, manual inspection methods consume significant manpower and material resources, and the repetitive nature of the work, coupled with subjective evaluations, makes pipeline defect assessment prone to errors. With the ongoing process of urbanization and the increasing need for regular pipeline inspections, the efficiency and quality of manual defect detection through visual information cannot be guaranteed. There is an urgent need to develop efficient and intelligent detection methods to meet the demand for long-term pipeline detection and maintenance in cities [5].

The emergence and development of machine vision and artificial intelligence technology [6,7] have provided new ideas for the intelligent detection of pipeline defects. Early researchers utilized computer vision algorithms, support vector machines [8], and machine learning algorithms, such as random forest [9] and histograms of oriented gradients (HOGs) [10], for pipeline detection. Although capable of completing the defect detection task to some extent, these methods require substantial computing power for preprocessing and postprocessing, proving inefficient and falling short of the accuracy, real-time performance, and practical deployment demands of modern urban pipeline detection. Considering the practical scenario of defect detection in drainage pipelines, the use of target detection technology is a more suitable choice. The current mainstream defect detection algorithms can be broadly classified into two main categories: single-stage algorithms, such as SSD [11] and the YOLO series [12], and two-stage networks, such as the R-CNN [13] series, Fast R-CNN [14], and Faster R-CNN [15]. In recent years, numerous researchers have focused on employing various computer vision technologies to achieve the automated detection of defects in urban drainage pipes. Duran et al. [16] proposed a method that leverages neural networks and image feature extraction. By integrating pipe image data collected from cameras and laser profiler sensors, the approach significantly enhances defect detection performance in low-light pipe environments. Similarly, Guo et al. [17] introduced a detection method based on the analysis of differences between image frames, demonstrating rapid defect detection capabilities to a certain extent. Cheng et al. [18] utilized CCTV to collect 3000 images of drainage pipes for training and verification and adopted the Faster R-CNN algorithm for detection, achieving an average accuracy of 83% and a detection speed of 9.434 FPS. Li et al. [19] employed pipeline photographs collected via CCTV and QV and, based on the deep learning method of two-stage learning, improved the defect detection mAP by 1.1% by strengthening the region proposal network (RPN) and multilayer global feature fusion. Kumar et al. [20] gathered 2100 sewer images via CCTV for training and verification. They adopted a two-step detection strategy, initially classifying and subsequently using YOLO for fracture detection, achieving an average accuracy of 71%.

In practical applications, the YOLO model series stands out for real-time detection tasks due to its balance between speed and accuracy. For instance, Tan et al. [21] enhanced an automatic sewer defect detection approach using YOLOv3, where the improvement involves a loss function, bounding box prediction, and network architecture. Yin et al. [22] proposed real-time automatic detection of sewer defects via the YOLOv3 network, which uses a dataset of 4056 instances to implement six types of defects (breaks, holes, sediments, cracks, fractures, and roots), achieving an mAP value of 85.37%. Oh et al. [23] incorporated the convolutional block attention module (CBAM) into the YOLO framework to enhance detection accuracy. Kumar et al. [24] conducted a comparative analysis of SSD, YOLO, and Faster R-CNN for identifying root invasion and sewer sediment issues, highlighting YOLO’s advantages in balancing detection speed and accuracy. As small embedded devices gain increasing use in engineering applications, there is growing concern among experts regarding the balance between lightweight model design and detection accuracy. Various deep learning methods have been proposed to address different needs. However, for small embedded devices, the detection accuracy and model size need to be further balanced and improved.

In response to these problems, researchers have proposed methods of model compression and pruning, which aim to reduce the computing and storage overhead of models based on the target premise of the existing dataset. Zhang et al. [25] proposed a Slim-YOLOv3 model to achieve real-time target detection through UAV image collection by pruning redundant channels and network layers. Wu et al. [26] presented a YOLOv4 deep learning algorithm based on channel pruning for the rapid detection of apple blossoms. This pruning method effectively reduces the model’s size and inference time without compromising accuracy. Situ et al. [3] proposed a YOLOv5s pipeline defect detection method based on channel pruning, which simplifies the network by reducing the number of channels. Zhang et al. [27] introduced a channel pruning-based compression technique for YOLOv7, which significantly reduces the model’s parameters, although this results in a slight decrease in detection accuracy.

Two-dimensional detection images face certain limitations in urban pipeline inspection, primarily due to their lack of depth information, which hinders their ability to fully represent the actual condition of pipelines [28]. This challenge is further exacerbated in low-light conditions, where target detection becomes significantly more difficult [29]. Moreover, urban pipelines feature curved surfaces, and 2D imaging technology fails to accurately capture their 3D morphology. Consequently, achieving precise defect measurement and quantitative evaluation solely based on 2D images remains challenging. To address this issue, the application of 3D point cloud technology in pipeline inspection provides an innovative solution [30]. By capturing the three-dimensional coordinate information of object surfaces, 3D point cloud technology can construct realistic 3D models, enabling a comprehensive and accurate representation of pipelines. This approach offers strong support for the precise measurement and evaluation of defects. Previous studies have focused mainly on the use of 3D laser scanners or depth cameras to capture the depth maps and 3D point clouds of various objects. For example, Tan et al. [31] used LiDAR to diagnose pavement defects. In three-dimensional sewer scenarios, researchers have employed various techniques to reconstruct urban drainage pipes in 3D. Current related studies primarily focus on the use of laser or sonar technology for three-dimensional pipeline scanning. Lepot et al. [32] introduced an unbiased and high-precision laser profilometer, which was tested on various sewer defects and demonstrated significantly improved measurement accuracy compared to traditional CCTV systems. Additionally, Bahnsen et al. [33] employed depth cameras to obtain precise point cloud representations of PVC pipes. However, current 3D point cloud acquisition devices are not only costly but also face limitations due to the minimum distance required between the 3D sensor and the target [34]. Furthermore, 3D segmentation methods that directly process point clouds tend to be computationally intensive and resource-heavy [35]. In comparison, image acquisition via CCTV systems has gained widespread adoption. Compared to the use of LiDAR, RGB cameras are more affordable, and the process of image acquisition is simpler; this image-based approach offers greater economic and practical benefits for real-world applications [34,36].

Zhang et al. [37] proposed a 3D reconstruction method for urban drainage pipes based on multiview image matching using a low-cost panoramic camera, offering a practical and efficient solution for pipeline detection. Fang et al. [38] proposed a sewer inspection framework based on computer vision technology that uses RGB image data collected by a floating capsule robot to achieve image defect instance segmentation, equipment localization, and 3D model reconstruction. Ma et al. [39] developed an improved 3D analysis and modeling platform for sewage pipelines. This platform identifies common issues, such as misalignment, blockages, and cracks, via 2D image recognition. It processes image data from multiple viewpoints to generate depth maps and employs a 2D-to-3D conversion algorithm, successfully creating virtual replicas of two real pipeline segments. Wang et al. [40] focused on the automatic classification and segmentation of 3D sewer pipelines. By refining the neural network architectures and learning strategies used in existing inspection processes, the accuracy of defect identification was improved, particularly in handling 3D point cloud data.

Existing 2D image-based detection methods for pipeline defects often focus on improving a single aspect, such as increasing detection accuracy or achieving a lightweight model. However, finding a balance between these two aspects can be challenging. YOLOv7-tiny performs well in terms of speed and model compactness, yet there is potential for further optimization. In practical engineering applications, detailed depth information maps can be generated from multiview pipeline images, enabling the construction of a 3D model to accurately represent the overall defect structure. The 3D model allows for the calculation of key parameters, such as defect size and volume, facilitating a more scientific assessment of the defect’s severity. This approach provides robust support for the measurement and evaluation of pipelines.

**Figure 1:**
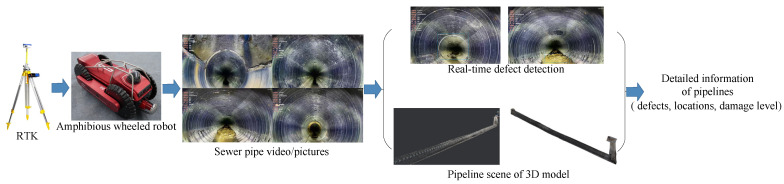
Pipeline defect detection and 3D scene reconstruction framework.

This paper combines the advantages of 2D image detection algorithms and 3D point cloud technology and proposes a computer vision-based inspection framework using pipeline robots for sewer defect detection. The main contributions of this article are summarized as follows:

1. A framework for drainage pipe defect detection and 3D reconstruction is proposed to obtain comprehensive pipe condition information. This framework is illustrated in Figure 1.
2. A Sewer-YOLO-Slim detection model is proposed for the automatic detection of urban drainage pipe defects, and the proposed model is deployed on pipe robot equipment.
3. The YOLO model is optimized in three key areas: enhancing the backbone network and neck network, integrating an attention mechanism within the detection head (DyHead), and pruning the proposed model to achieve a lightweight model design.
4. The framework implements the positioning of pipeline inspection robots, the reconstruction of realistic 3D sewer scenes, and measurement functionality. This enables drainage pipeline condition data to be collected more comprehensively and presented in a clear and intuitive manner.

The structure of this paper is organized as follows. Section 2 provides an in-depth explanation of the data acquisition, the Sewer-YOLO-Slim algorithm, and the computer vision methods utilized for 3D reconstruction. Section 3 outlines the experimental setup and configuration details. Section 4 describes the experimental outcomes for defect detection and 3D modeling, and Section 5 presents our conclusions.

## 2. Materials and Methods

### 2.1. Amphibious Wheeled Robot

**Figure 2:**
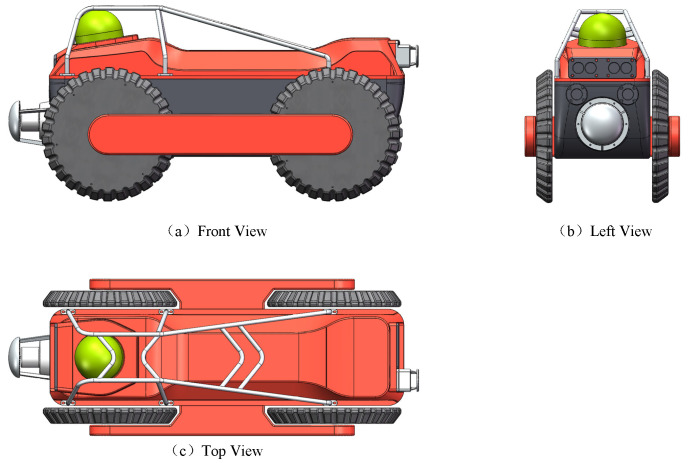
Three views of the amphibious wheeled pipe inspection robot.

To achieve efficient defect detection in urban drainage systems, an amphibious wheeled pipe inspection robot device is designed. Three views of the amphibious wheeled pipe inspection robot are shown in Figure 2.

**Figure 3:**
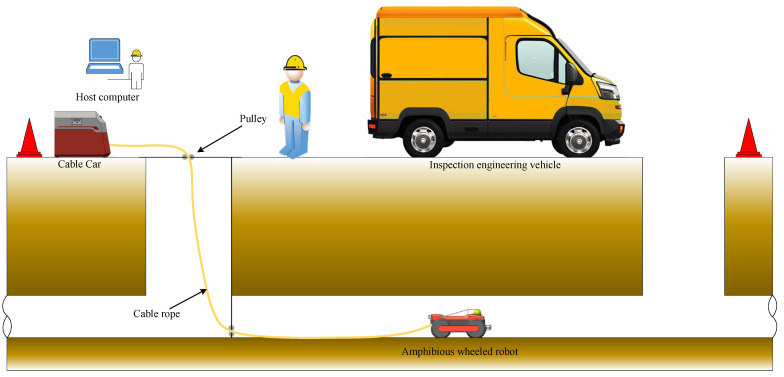
Pipeline detection and data collection process.

The amphibious wheeled pipe inspection robot utilized in this study is equipped with a high-resolution CMOS non-infrared camera, which meets the IP68 waterproof standard. The camera supports horizontal rotation from 0 to 360 degrees and vertical rotation from 0 to 180 degrees. Additionally, the robot is fitted with various sensors, including an inertial measurement unit (IMU), enabling real-time video data capture as it moves along the inner wall of the pipe. Figure 3 illustrates the detailed process of data collection and inspection inside the urban sewer. Using real-time kinematic (RTK) instruments, the GPS positioning of pipeline inspection shafts accurately records the location data of the inspection wellhead. The amphibious wheeled inspection robot is then deployed in the pipeline. The developed robot exhibits stable movement capabilities, enabling continuous data acquisition along the axial direction of the pipe. Due to the lack of wireless signals in underground sewers, the integration of multi-sensor information plays a crucial role in robot positioning, defect detection, and the accurate representation of world coordinate positions in 3D modeling.

### 2.2. Urban Sewer Defect Image Database

**Figure 4:**
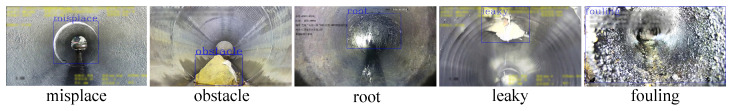
Five types of defect examples and labels.

An urban sewer defect image database (USDID) was created to assess the efficacy of the proposed target detection algorithm. The original image set was evaluated, and five prevalent types of pipeline defects were selected as identification targets for the detection model. These defect types include misplacements, obstacles, roots, leaks, and fouling, as defined in [41]. Figure 4 shows the defects and labels. Table 1 presents the defect data in the USDID.

### 2.3. YOLOv7-Tiny Algorithm

**Figure 5:**
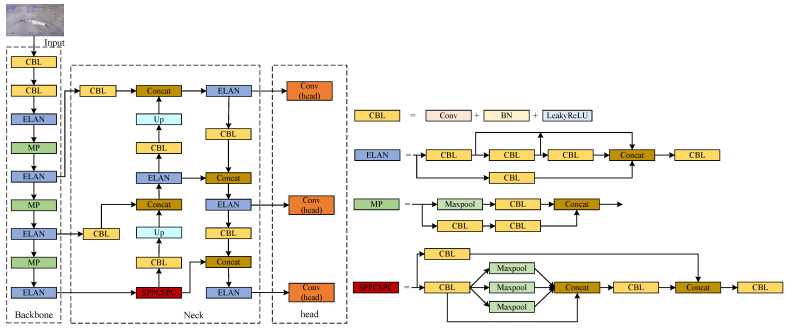
YOLOv7-tiny network structure.

YOLOv7 [42] is the seventh-generation version of the YOLO target detection algorithm. YOLOv7-tiny [43], depicted in Figure 5, is a simplified variant of YOLOv7 comprising three main components: the backbone network, neck network, and prediction head. In the backbone section, the E-ELAN structure is replaced with the more concise ELAN; MP performs only pooling downsampling; the neck part adopts a PANet structure for feature aggregation; and in the head section, REPConv is substituted with a standard Conv to adjust the number of channels. Compared to YOLOv7, YOLOv7-tiny sacrifices some accuracy but excels in terms of its lightweight design. However, YOLOv7-tiny still presents areas for further optimization. The ELAN network employed in the backbone exhibits a complex structure and many parameters. The use of ELAN networks in the neck may introduce redundancy in feature extraction. This paper aims to enhance YOLOv7-tiny’s performance by proposing a more lightweight solution to reduce the number of parameters and computations.

**Figure 6:**
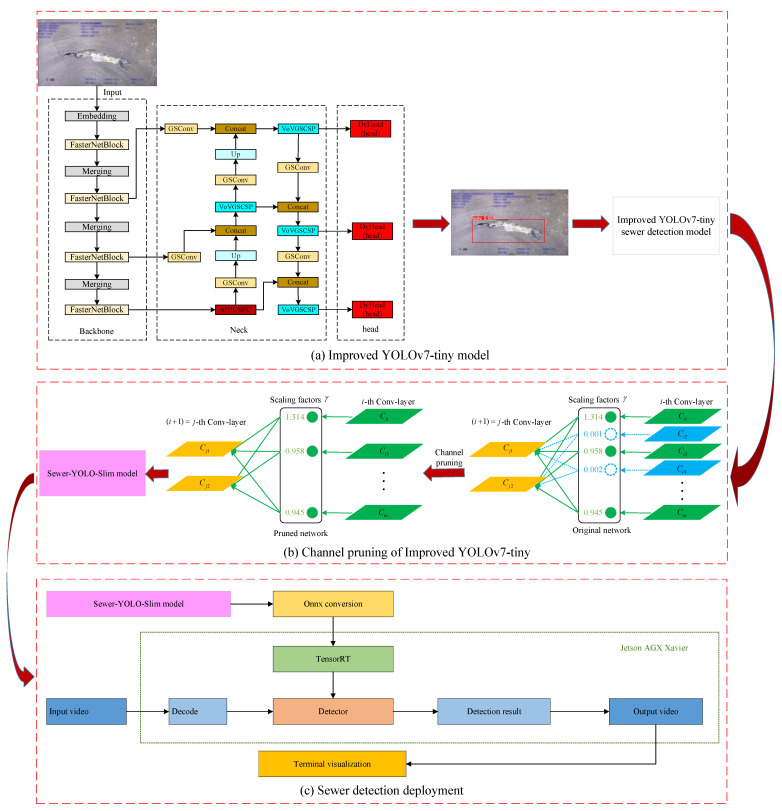
The technical process of the proposed method. (**a**) The improved structure of YOLOv7-tiny. (**b**) Channel pruning is applied to the improved model. (**c**) The process of Sewer-YOLO-Slim being deployed on the edge detection device.

This article makes the following improvements to YOLOv7-tiny: First, the lightweight FasterNet is employed as the backbone network, and the effectiveness of these modifications is experimentally validated. Second, in the neck part of the model, based on the lightweight slim neck idea, the GSCConv and VoVGSCCSP modules are introduced to reduce parameters and perform feature aggregation to obtain richer features. In the head part, the DyHead target detection head, incorporating the attention mechanism, is utilized to improve spatial perception capabilities and detection accuracy. The detailed structure of the proposed improved YOLOv7-tiny is shown in Figure 6a. Subsequently, channel pruning is applied to the improved YOLOv7-tiny model to reduce channels that have little impact on detection accuracy while retaining important feature information, making the model more lightweight and improving detection efficiency. The channel pruning operation is illustrated in Figure 6b. To address the limitations of high-end hardware, the pruned model, named Sewer-YOLO-Slim, is transferred to edge development devices for rapid onsite data processing, and the model is converted to the ONNX format for further optimization. This enables TensorRT acceleration, allowing for deployment on edge detection devices to achieve real-time detection in field applications with the pipeline inspection robot. The deployment process of the pruned model is illustrated in Figure 6c.

### 2.4. Sewer-YOLO-Slim Model Construction

#### 2.4.1. FasterNet Algorithm

**Figure 7:**
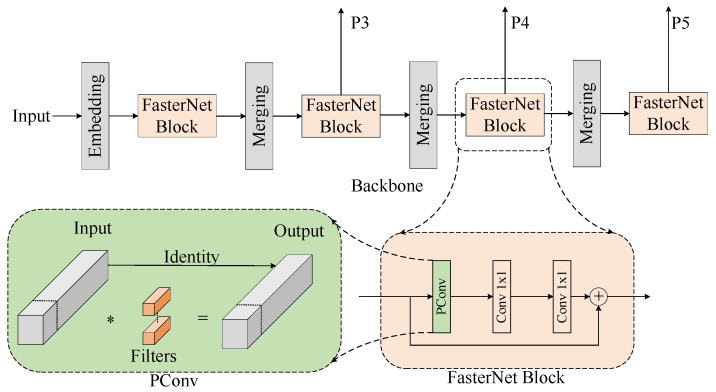
The architecture of FasterNet and its improvements.

FasterNet [44] is a lightweight network model released by the Hong Kong University of Science and Technology team in March 2023. It exhibits broad applicability and operates efficiently on edge devices. This model is built upon the PConv operation, which reduces redundant information in the feature map and systematically applies conventional convolution to some of the input channels while leaving the other channels unchanged. This operation effectively reduces the complexity and the number of parameters. FasterNet has multiple models, including FasterNet-T0/1/2 and FasterNet-S/M/L. Given the lightweight requirements for pipeline defect detection, FasterNet-T0 is chosen as the backbone network for our model. This selection significantly reduces the number of parameters. Figure 7 illustrates the FasterNet network structure and its improved components. The primary architecture consists of four levels, each comprising multiple FasterNet blocks, with PConv being the main operation.

#### 2.4.2. GSCConv and VoVGSCCSP Modules

**Figure 8:**
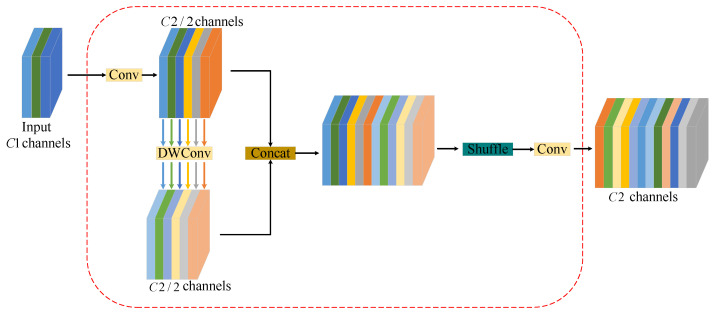
The structure of the GSCConv module.

For the neck part, this article replaces the standard convolution with the improved GSCConv module for both upsampling and downsampling operations. By adding convolution, the network’s feature extraction capability is enhanced. Li et al. [45] proposed the GSConv and VoVGSCSP modules in the slim neck structure. The structure of the enhanced GSCConv module is illustrated in Figure 8. Assuming that the number of input channels is C1 and the number of output channels is C2, the standard convolution reduces the number of channels to C2/2. This is followed by a depthwise separable convolution, where the number of channels remains constant. After two convolutions, splicing and shuffling operations are performed. The shuffling operation reorganizes the channel information, enhancing the fusion of multifeature information and improving the expressive capacity of image semantic information.

**Figure 9:**
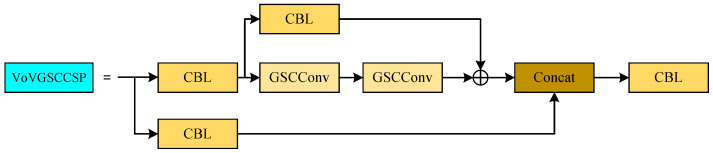
The structure of the VoVGSCCSP module.

The ELAN is substituted with the improved VoVGSCCSP module for cost-effective detection calculations. The specific VoVGSCCSP structure is illustrated in Figure 9. The two convolutions preceding the Concat layer employ the GSCConv module, which efficiently reduces the number of model parameters while maintaining detection accuracy.

#### 2.4.3. DyHead Module

**Figure 10:**
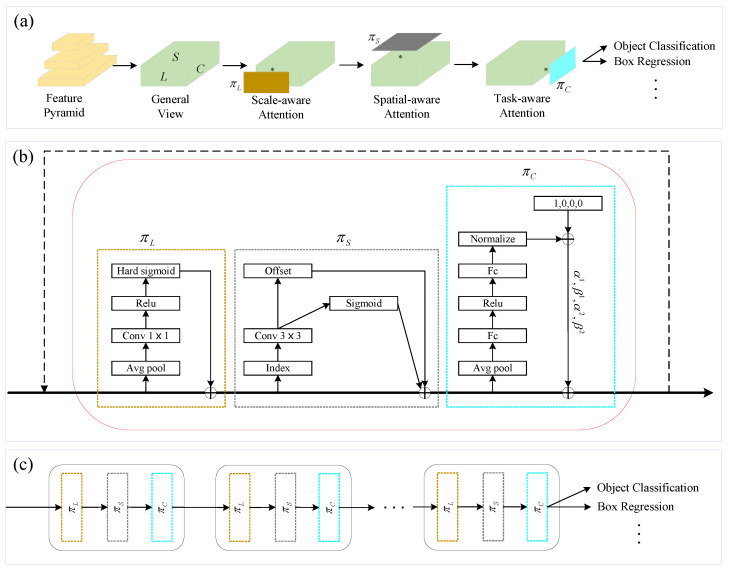
The DyHead structure. (**a**) Description of a three-dimensional tensor from a general view, where L, C, and S represent the number of layers, channels, and the height and width of the feature map, respectively. (**b**) Three attention mechanisms (scale-aware, spatial, and channel) are applied in sequence. (**c**) The specific application of DyHead in the detection framework.

The urban pipeline defect dataset has a complex background, encompassing different materials, pipe diameters, and multiple defect categories. Consequently, the detection algorithm must possess a comprehensive understanding of the pipeline. DyHead [46] introduces a novel dynamic head framework to integrate head and attention mechanisms in object detection tasks. This approach significantly improves target detection performance by integrating scale, spatial, and task attention mechanisms using a multihead self-attention approach within the feature layer. The detailed structure of DyHead is illustrated in Figure 10.

DyHead achieves the fusion of scale, spatial, and task awareness by integrating multiple self-attention mechanisms across feature layers, thereby enhancing the performance of the target detection head. Scale awareness focuses on the size of the pipeline defect range and enhances features on appropriate layers by learning the relative importance between feature layers. This adaptation to defects of different sizes improves detection robustness. Spatial perception contributes to increased detection accuracy by learning spatial differences between defects at various locations within the defect image, thus more effectively capturing specific location information. Task awareness processes pipeline defect feature data across different channels, guiding the feature channels to identify diverse defects, processing varied feature information, and improving the generalizability of detection. The synergy of these three attention mechanisms enables DyHead to utilize the relationships among feature layers comprehensively, effectively enhancing the detection of pipeline defects.

#### 2.4.4. The Design of Network Pruning

**Figure 11:**
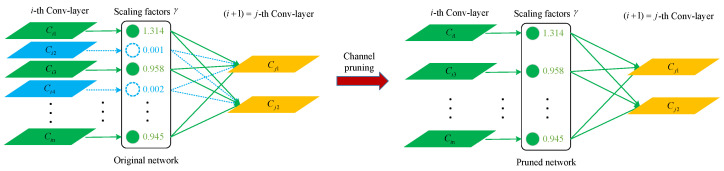
Schematic diagram of the channel pruning algorithm.

The improved YOLOv7-tiny model accurately identifies pipeline defects; however, further optimization opportunities exist. This paper employs model pruning to reduce structural complexity, making the model deployable on devices with limited computing power. As illustrated in Figure 11, the scaling factor γ of the batch normalization layer is utilized to assess channel importance. Important channels (green channels) are retained, whereas unimportant channels (blue channels) are removed. The pruning decision requires a comprehensive consideration of the distribution of the γ factors and the channel pruning rate.

When structural sparsity is incorporated into the improved model, the coefficient γ of the batch normalization (BN) layer is used to evaluate channel importance. Channels with lower importance in the improved model are eliminated according to the predetermined pruning rate, resulting in a more storage-efficient model. After channel pruning, meticulous fine-tuning recovery is conducted to address any potential degradation in accuracy, optimizing the pruned model for compact performance. This process involves performance evaluation, pruning iterations, and the eventual achievement of lightweight network models. Notably, an excessively high pruning ratio can lead to a significant loss of certain channels, thereby reducing model detection accuracy. Therefore, the pruning rate must be selected carefully to strike a balance between model compactness and performance.

### 2.5. Robot Positioning and 3D Reconstruction

#### 2.5.1. Amphibious Wheeled Inspection Robot Positioning

**Figure 12:**
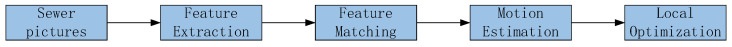
The localization process of the amphibious wheeled pipeline inspection robot.

The sewer environment is characterized by a range of challenging conditions, such as low lighting, water accumulation, and structural damage to pipes. In such environments, accurately determining the position and orientation of the robot via sensors can be a challenging task. Currently, CCTV inspection robots equipped with cameras are widely used for video capture and recording. Visual odometry (VO) technology [47] addresses these challenges by analyzing consecutive image frames to estimate the movement of the camera or robot. Given the unique nature of drainage pipelines, the positioning of the inspection robot can be considered a VO problem. Figure 12 illustrates the localization process of the pipeline inspection robot.

Feature Extraction: Scale-invariant feature transform (SIFT) is used to detect key points in images and generate feature descriptors [48]. This process identifies feature points in images that are highly distinctive and invariant to scale and rotation, making them suitable for matching across images with different viewpoints and scales.

Feature Matching: After the feature points of multiple images are obtained, feature point matching across images is performed. The nearest neighbor algorithm is used for feature matching, and then RANSAC is used to eliminate mismatches and optimize the matching results.

Motion Estimation: After feature point matching is completed, camera motion can be estimated via geometric methods. According to the projection relationship between 2D and 3D points in an image, the perspective-n-point (PnP) algorithm is used to solve the camera’s position and orientation [49]. The core formula for this problem is as follows:

(1) $$\lambda \left[\begin{array}{c} u_{i}\\ v_{i}\\ 1 \end{array}\right]=K\left[\begin{array}{cc} R & t \end{array}\right]\left[\begin{array}{c} X_{i}\\ Y_{i}\\ Z_{i}\\ 1 \end{array}\right]$$

where λ is a scaling factor. The 3D points $\left(X_{i},Y_{i},Z_{i}\right)$ in world coordinates are related to their 2D projection $\left(u_{i},v_{i}\right)$ in image coordinates through the camera’s intrinsic matrix K, rotation matrix R, and translation vector t.

Local Optimization: After the camera poses are estimated, bundle adjustment (BA) is applied to refine both the 3D coordinates and the camera poses [50]. BA achieves this by minimizing the sum of the squared projection errors between the observed image points and the corresponding projections of the 3D points.

The projection error is defined as

(2) $$\mathrm{error}=\sum_{i=1}^{n}{∥\mathrm{proj}\left(X_{i},R_{j},t_{j},K\right)-{\hat{x}}_{ij}∥}^{2}$$

where the function $\mathrm{proj}(X_{i},R_{j},t_{j},K)$ represents the projection of the 3D point $X_{i}$ onto the image plane through camera *j*; $R_{j}$ and $t_{j}$ represent the rotation matrix and translation vector of camera *j*, respectively; and *K* is the camera’s intrinsic matrix. The term ${\hat{x}}_{ij}$ is the observed 2D point corresponding to the projection of $X_{i}$ in the image.

#### 2.5.2. Three-Dimensional Reconstruction of the Pipe Scene

**Figure 13:**
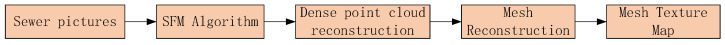
Three-dimensional reconstruction process of the pipeline scene.

The purpose of positioning a sewer amphibious wheeled inspection robot is to obtain images of the internal structure of the drainage system as well as the robot’s location, which is critical for constructing a 3D model of the pipeline network. Figure 13 illustrates the workflow of the entire 3D model reconstruction process.

The process involves various stages, from image acquisition to the generation of a textured 3D model, integrating several computer vision techniques and graphical processing methods.

SFM Algorithm: The structure from motion (SFM) [51] recovers the 3D structure of an object from a 2D image with multiple views. This technique identifies and matches key points across images and uses the relative motion between the camera positions to estimate the 3D structure of the scene.

Dense Reconstruction: Building on the initial sparse point cloud obtained from SfM, dense reconstruction algorithms [52] create a more detailed 3D representation by calculating depth information for every pixel in the images.

Mesh Reconstruction: The dense point cloud is converted into a mesh by creating a continuous surface using triangulation methods [53].

Texture Mapping: To make the 3D model look more realistic, the color information of the image is mapped to the mesh surface.

With the help of open-source 3D modeling tools, such as the open-source multiview geometric reconstruction library (OpenMVG) [54] and the multiview stereo reconstruction toolkit (OpenMVS) [55], the construction from raw image data to the final 3D scene is completed.

## 3. Configuration and Evaluation

### 3.1. Experimental Configuration

The software used in this study included PyTorch version 1.11.0, Cuda version 11.3, and Python version 3.8, running on a computer with the Ubuntu 18.04 operating system. The CPU was an Intel(R) Xeon(R) Gold 5320 CPU@ 2.20 GHz, by Intel Corporation, headquartered in Santa Clara, United States, and the system was equipped with 64 GB of running memory. The graphics card used was the NVIDIA GeForce RTX 3090 (24 GB), produced by NVIDIA Corporation, also based in Santa Clara, United States. The algorithm training parameters are detailed in Table 2, while the pruning experiment parameters are specified in Table 3.

Following pruning, the enhanced model, Sewer-YOLO-Slim, was deployed for onsite testing on the embedded AI terminal device EA-B400. The EA-B400 is an edge development device produced by Lianbao (Hefei) Electronics Technology Co., Ltd., located in Hefei, China. It is equipped with the NVIDIA Jetson AGX Xavier core module manufactured by NVIDIA Corporation. This core module boasts a computing power of up to 32 TOPS and an I/O performance of up to 750 Gbps, making it highly suitable for real-time onsite pipeline data processing. The EA-B400 was assembled into the cable car of the crawling robot’s retracting and unwinding system and integrated with sensors and data acquisition systems to achieve onsite pipeline data collection and processing.

### 3.2. Evaluation Metrics

Target detection evaluation metrics usually include precision, recall, average precision (AP), mAP, and inference time. The calculation formulas are as follows:

(3) $$\mathrm{Precision}=\frac{\mathrm{TP}}{\mathrm{TP}+\mathrm{FP}}$$

(4) $$\mathrm{Recall}=\frac{\mathrm{TP}}{\mathrm{TP}+\mathrm{FN}}$$

(5) $$\mathrm{AP}=\int_{0}^{1}p\left(r\right)\;dr$$

(6) $$\mathrm{mAP}=\frac{\sum_{i=1}^{K}{\mathrm{AP}}_{i}}{K}$$

True positives (TP) refer to the correctly identified positive samples, while false positives (FP) denote instances where the model incorrectly predicts positive results. False negatives (FN) signify cases where the model incorrectly predicts non-defects as negative results. The AP can be expressed as the area under the precision–recall (P/R) curve. K is the number of categories for the detected target, and the average of the K categories is taken to obtain the mAP.

## 4. Experiment and Results

### 4.1. Sewer Defect Detection

#### 4.1.1. Backbone Network Experiment

In pursuit of the goal of lightweight pipeline defect detection, this paper explored the impact of replacing the YOLOv7-tiny backbone network with five different mainstream lightweight models. These networks included GhostNet, ResNet18, EfficientViT_M0, MobileNetV3-Small, and the modified FasterNet-T0. The experiment focused solely on modifying the backbone network structure, and the comparison results are presented in Table 4.

The results indicate that, compared with YOLOv7-tiny, employing the lightweight model backbone GhostNet, ResNet18, EffcientViT-M0, and MobileNetV3 networks led to a reduction in parameters but at the cost of varying degrees of accuracy loss and longer training times. The use of the FasterNet-T0 backbone network resulted in a 1.4% increase in the mAP of the detection network, along with reductions in model parameters and volume and an acceleration in training speed, and the network still has potential for further optimization.

#### 4.1.2. Ablation Experiment

To assess the effectiveness of each improvement in YOLOv7-tiny, an ablation experiment was devised using the same datasets and identical hardware environment configurations. The specific design of the ablation experiment is outlined below.

The designs of the different solutions are shown in Table 5, where “✓” indicates that the corresponding improvement method was used, and “×” means that it was not used. The ablation experiment results are shown in Table 6.

From Table 6, it is evident that the precision, recall, and mAP values of Plan 1 significantly increased compared to those of Plan 0, indicating the effectiveness of FasterNet-T0 as the backbone network in improving recognition accuracy. Plan 2, which incorporates the GSCConv and VoVGSCCP structures, contributed significantly to model compactness by reducing the model’s parameters and computational complexity. Plan 3 introduces DyHead, an attention-based mechanism, resulting in a substantial improvement in accuracy. Through the ablation experiments, from Plan 0 to the final Plan 4, the effects of each improvement were demonstrated, albeit at the cost of increased algorithm inference time. The proposed method in the final scheme achieved increases of 1.8%, 3.1%, and 1.8% in precision, recall, and mAP values, respectively. Furthermore, the reductions in model size, parameters, and FLOPs were 17.1%, 18.1%, and 31.8%, respectively. These results indicate that the optimizations applied throughout this work successfully enhanced the model’s performance.

#### 4.1.3. Channel Pruning Experiment

After the weight file of the improved YOLOv7-tiny model was optimized, the channels were pruned. To prevent potential overpruning, the channels were tested at pruning rates of 40%, 50%, 60%, 70%, and 80%, respectively. The goal was to assess the pruning effect and determine the most suitable pruning rate. Table 7 presents the comparative results after channel pruning and fine-tuning of the improved model.

**Figure 14:**
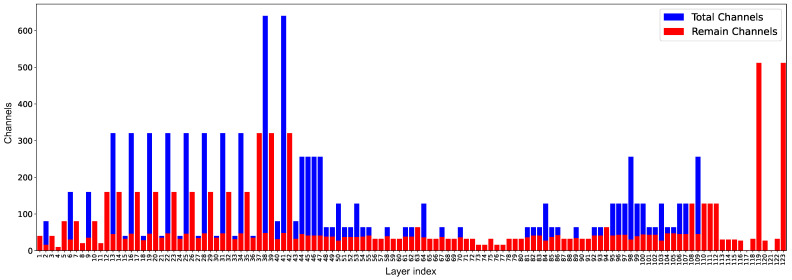
Channel changes before and after pruning.

After the channel pruning operation, the detection accuracy of the model decreased. At a pruning rate of 50%, a good balance was achieved between pruning accuracy and model size, with an average accuracy (mAP) loss of only 0.3%. When the pruning rate was 60%, more important channels and convolutional layers were pruned, resulting in a notable 4.4% decrease in detection accuracy. In summary, a pruning rate of 50% allows for model size compression while maintaining the original detection performance. Figure 14 illustrates the change in the channels after the pruning rate was set to 50%. The figure shows that the pruning algorithm effectively reduced the number of channels.

#### 4.1.4. Comparison of Different Detection Algorithms

This paper conducted a comparative analysis against mainstream two-stage and single-stage target detection algorithms under the same experimental conditions. The results are summarized in Table 8.

The proposed Sewer-YOLO-Slim achieved an optimal balance between model size and accuracy, with a smaller size, fewer parameters, and fewer FLOPs, while maintaining high detection precision. Compared with the Faster-RCNN and SSD models, the improved algorithm has greater advantages in terms of model size and mAP for target defect recognition. Furthermore, in comparison to the original YOLOv5l, YOLOv4, and YOLOv3 models, the mAP values increased by 4.0%, 7.7%, and 5.6%, respectively. Compared with YOLOv7, YOLOv8n, YOLOv9s, YOLOv10n, and YOLOv11n, some metrics for the Sewer-YOLO-Slim proposed in this paper were slightly lower. However, considering the model size, parameters, and computational load, the proposed model achieved optimal performance, especially after the pruning operations. The algorithm offers obvious advantages for small embedded devices with limited space.

Compared to the original YOLOv7-tiny, the improved algorithm, after pruning, resulted in a 65.9% reduction in FLOPs, a 60.0% reduction in the number of parameters, a 60.2% reduction in model size, and a 1.5% increase in mAP.

**Figure 15:**
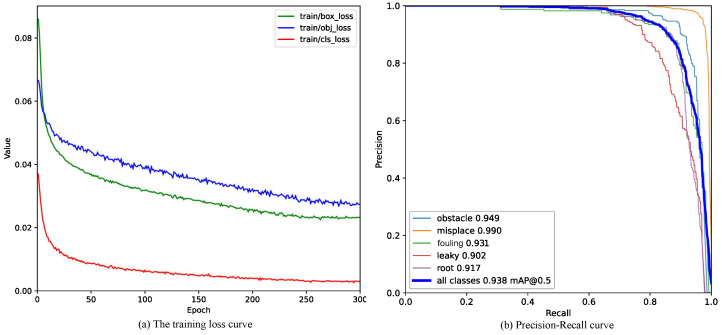
The improved YOLOv7-tiny’s training loss and the precision–recall curve. (**a**) Loss curves at three different stages during the entire training process of the improved model. (**b**) Precision–recall curve of the improved algorithm.

Figure 15a shows that the loss function curve converged quickly during the training process, indicating that the improved algorithm has strong stability. Figure 15b shows that the improved algorithm achieved high accuracy in misplacement detection but faced challenges with leakage and roots.

**Figure 16:**
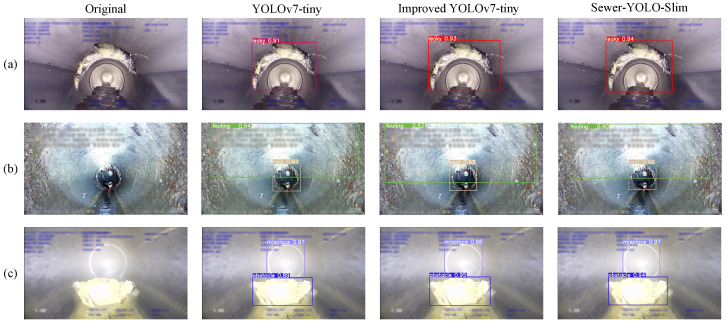
Comparison of different models on the USDID dataset. (**a**) Detection of leaks. (**b**) Detection of misplacements and fouling. (**c**) Detection of obstacles and misplacements.

Figure 16 presents a comparison of YOLOv7-tiny, the improved YOLOv7-tiny, and the improved YOLOv7-tiny after pruning, named Sewer-YOLO-Slim. The proposed algorithm performed better in terms of the defective target accuracy and target position detection frames.

In summary, the algorithm presented in this paper demonstrated its ability to achieve high detection accuracy while maintaining a lightweight design, thus confirming the practicality and advantages of the proposed approach.

#### 4.1.5. Edge Deployment Experiment

The proposed Sewer-YOLO-Slim has a computational load of 4.5 GFLOPs and 2.41 M parameters. It was transferred to the embedded AI terminal device EA-B400 for onsite testing. Comparative experiments involving different devices are presented in Table 9, illustrating the impact of converting the model parameters of Sewer-YOLO-Slim to the fp16 format using TensorRT. Sewer-YOLO-Slim was deployed on the EA-B400 and achieved efficient onsite pipeline detection through model quantization, TensorRT serialization, and hardware acceleration. This deployment is suitable for edge computing applications in actual pipeline detection, aiming for inference acceleration. The quantized fp16 model maintained a high mAP of 92.7% and an inference speed of 15.3 ms/image. Therefore, TensorRT is instrumental in the real-time detection of pipeline defects using deep learning solutions for edge devices.

**Figure 17:**
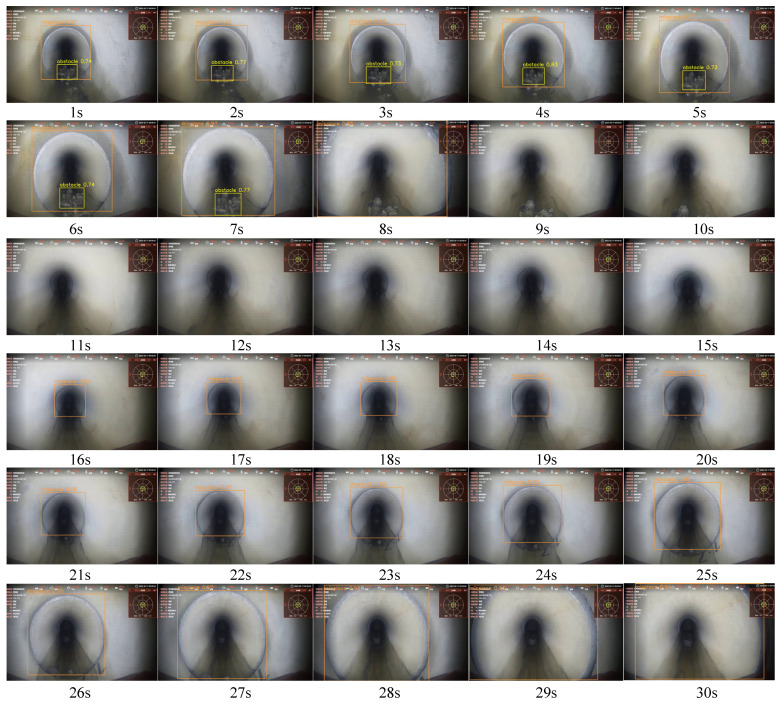
Onsite video inspection results.

In practical detection scenarios, where a video consists of consecutive frames showing the same defect over several seconds, two frames per second can be extracted for inspection. This approach overcomes the low speed associated with the limitations of edge device computing power. To validate the efficiency of pipeline video defect detection, a video of a real pipeline was inspected; the frames in the video are shown in Figure 17. The image acquisition speed was 10 FPS. The video included one obstacle and two misplacement defects. In most frames, defects were correctly identified and annotated. However, in the frames at 9 s and 10 s, the obstacles were incomplete and too small to be recognized. The experimental results indicate that the detection accuracy and efficiency meet the requirements for pipeline defect detection.

### 4.2. Amphibious Wheeled Robot Positioning and 3D Reconstruction

#### 4.2.1. Data

To validate the effectiveness and practicality of the proposed method, video data of a PVC corrugated drainage pipe were selected from a segment located between two concrete wells in a park in Hefei, China. The video was approximately 6 min long with a resolution of 2560 × 1440 pixels. The straight-line distance between the two wells was 18.2 m, and the sewer pipe was buried at a depth of 2.1 m. This geographical setting not only represents the typical characteristics of urban underground infrastructure but also offers a challenging environment in which to test the performance of the proposed method in a real-world application.

#### 4.2.2. Robot Positioning

**Figure 18:**
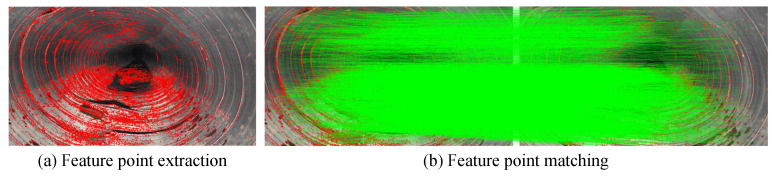
SIFT feature extraction and matching effect diagram.

In the process of using multiview images for positioning the inspection robot, the SIFT algorithm was employed to extract feature points from the images. Figure 18a shows the results of SIFT feature extraction, whereas Figure 18b shows the feature matching between two consecutive images of the pipeline. Both the SIFT feature extraction and the feature matching between adjacent images exhibit a high degree of accuracy. These precise feature points and matches provide a robust foundation for subsequent 3D reconstruction, ensuring a more accurate and detailed model of the pipeline.

**Figure 19:**
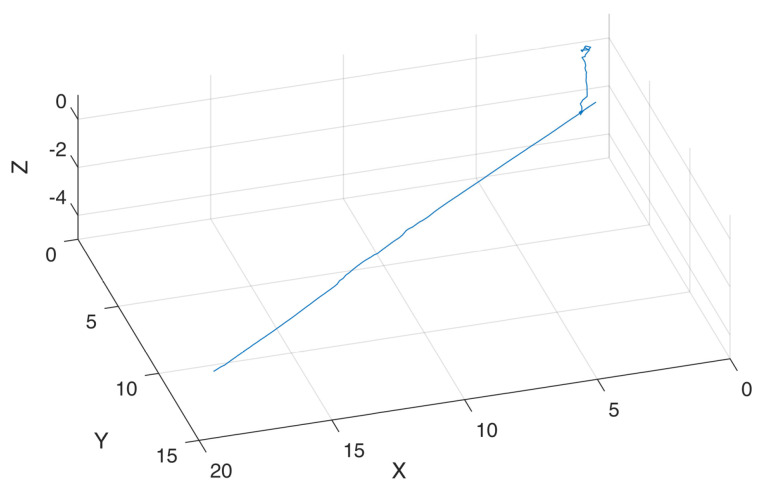
Motion trajectory tracking of the amphibious wheeled robot.

**Figure 20:**
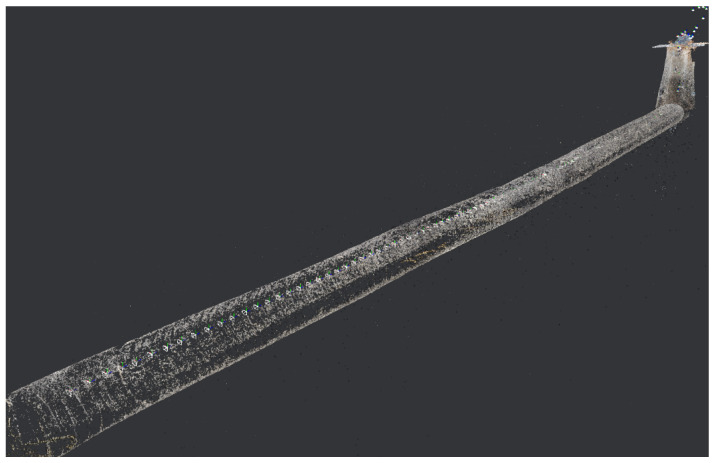
Positioning of the amphibious wheeled robot during image collection.

The results presented in Figure 19 illustrate the robot’s positional changes throughout the entire pipeline, whereas Figure 20 provides a detailed output of the camera positioning. The robot’s movement trajectory between the two inspection wells was nearly a straight line, indicating that the robot traveled along an almost linear path within the drainage pipeline. This straight-line movement not only simplifies subsequent data processing and analysis but also facilitates a more efficient interpretation of the pipeline’s condition.

#### 4.2.3. Three-Dimensional Construction

**Figure 21:**
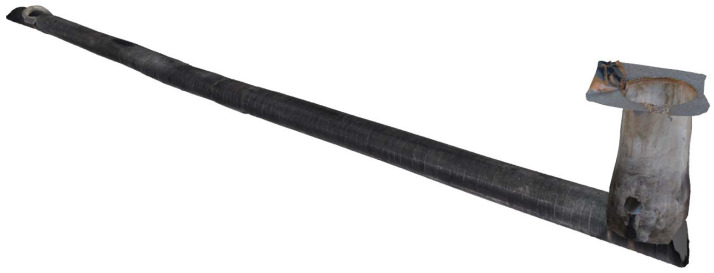
Textured 3D model of the sewer scene.

**Figure 22:**
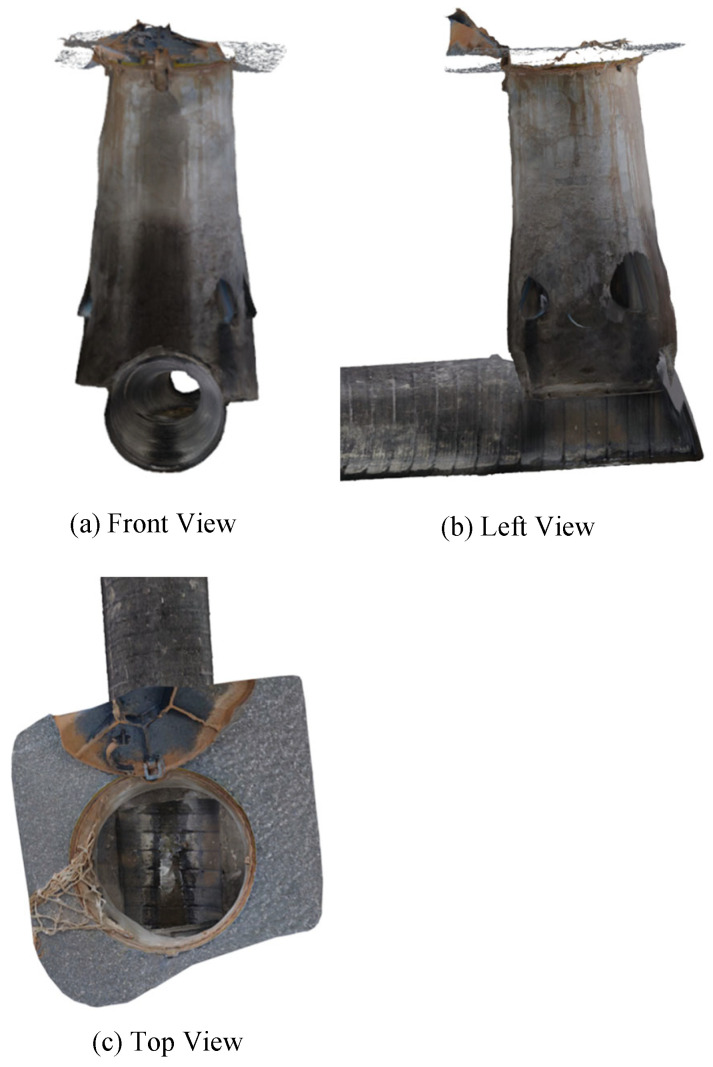
Three views of the textured 3D model of the inspection well.

**Figure 23:**
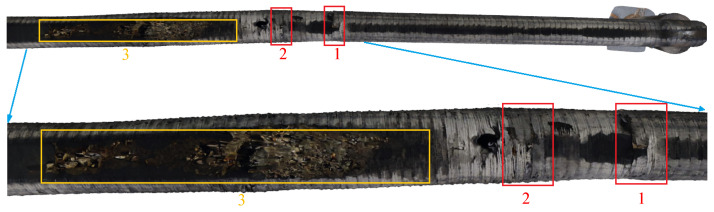
Three defects in the 3D model.

**Figure 24:**
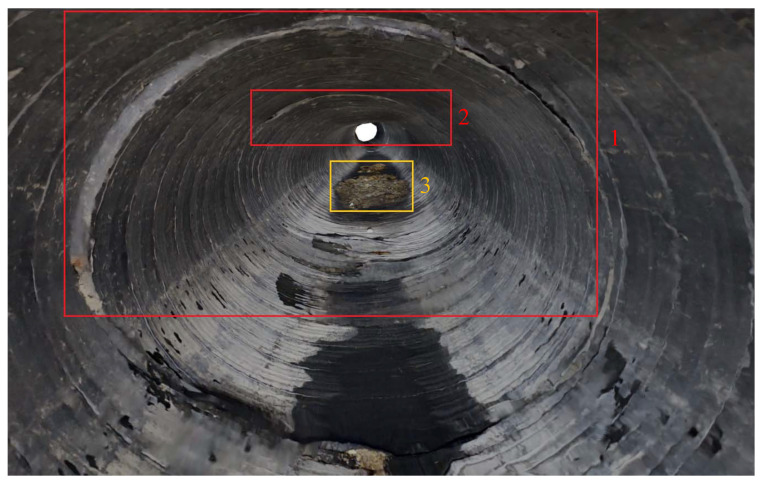
Three defects inside the 3D model.

The reconstruction results of the textured 3D model of the drainage pipeline and inspection well are shown in Figure 21 and Figure 22. These models not only offer a clear visual representation but also accurately replicate the real structural characteristics of the pipeline and inspection well. Figure 23 shows the observation results along the bottom of the pipeline, highlighting three locations (marked as 1, 2, and 3) where defects are potentially present. These areas of irregularity are particularly evident in the 3D model, providing valuable clues for further analysis of the pipeline’s condition. Delving deeper into the interior of the 3D pipeline model, as illustrated in Figure 24, reveals a more detailed view of the specific nature of these defects. This level of detail is crucial for developing effective repair strategies.

The 3D reconstruction results verify the effectiveness of the proposed method in practical applications and demonstrate its powerful capabilities in complex underground pipeline detection. This study provides reference materials for pipeline maintenance personnel and provides a scientific basis and technical support for future pipeline management and maintenance efforts. Monocular vision sensors face a scale ambiguity problem during 3D reconstruction, making it difficult to obtain the real dimensions of objects directly from images. To address this issue, the scale ratio was determined by measuring the corresponding real-world distances. Field surveys were conducted to gather actual data, including critical parameters such as the diameter of the inspection wells, the depth of the pipelines, and the distances between wellheads. By comparing these real-world measurements with the corresponding features in the 3D model, the model’s scaling factor could be accurately determined.

**Figure 25:**
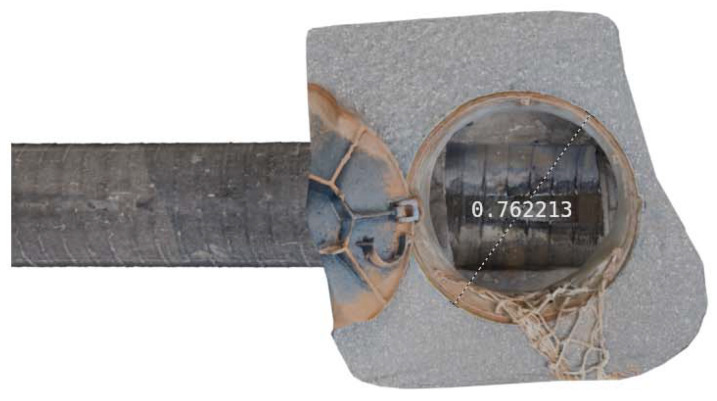
Inspection well bore diameter measurements.

Based on field survey data of the drainage pipeline, the straight-line distance between the two inspection wells was 18.2 m, with the burial depth of the drainage pipeline being 2.1 m below ground. The diameter of the surface inspection well was measured at 0.72 m. As shown in Figure 25, this diameter measurement served as the reference for calculating the absolute scale factor. Using the formula for scale factor calculation, the scale factor was determined to be 0.9446 m per unit, calculated as 0.72 m divided by 0.7622 units. This scale factor allowed for an accurate representation of real-world dimensions in the 3D model.

(7) $$Scale\;=\;\frac{real\;word\;length}{3D\;length}$$

Table 10 summarizes the results of the modeling process, indicating that the measurement errors for the pipe burial depth and pipe diameter were within a minimal range. Although the maximum error in measuring the pipe length reached 0.88 m, this level of deviation is not significant enough to impact excavation and repair work on the pipeline.

**Figure 26:**
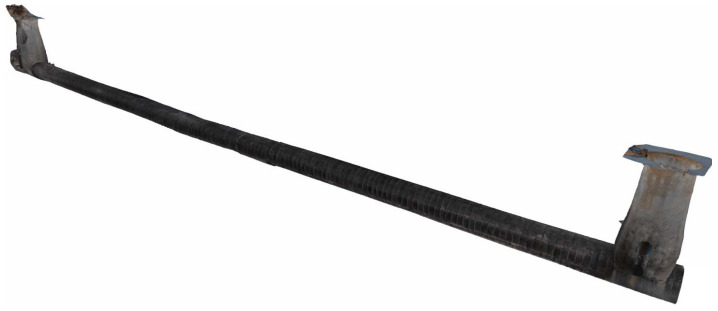
Textured 3D model of the drainage pipe based on bidirectional data.

To increase the accuracy of the three-dimensional reconstruction of drainage pipes, improvements were made based on existing methods. Specifically, the inspection robot was deployed from the opposite end of the pipe to capture additional images in the reverse direction. By integrating the image data collected from both directions, a more precise three-dimensional model can be generated. The textured 3D model of the drainage pipe resulting from this process is shown in Figure 26. Using the scale factor calculation Equation (7), the measurements for the bidirectional 3D model are summarized in Table 11.

When the inspection robot captured images while moving in only one direction, the constructed 3D model exhibited a maximum error of 0.88 m. In contrast, using bidirectional motion acquisition image data for modeling significantly improved the accuracy of the reconstruction, reducing the maximum error of the 3D model to 0.57 m. The error in the pipeline burial depth was eliminated, and the error in the pipeline diameter was reduced to 0.02 m. This approach effectively minimized errors while providing more comprehensive visual spatial coverage.

This finding offers a new method for achieving high-precision modeling and serves as a valuable reference for the structural design of inspection robots. For example, equipping a robot with additional cameras on its rear or top can help eliminate blind spots caused by fixed viewing angles. Moreover, objects that interfere with modeling, such as tail cables, can be removed from images during processing. By employing this multiangle image acquisition approach, a more complete and accurate representation of the internal space of the pipeline can be achieved, significantly enhancing the precision and comprehensiveness of 3D reconstruction.

**Figure 27:**
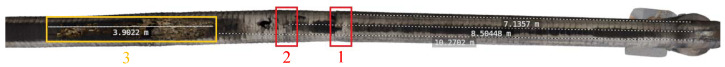
Defect location and measurement of the axial pipeline 3D model.

To further enhance the practicality of the 3D reconstruction model, the calculated scale factor was applied within Blender (the software used version is 4.2). This allowed for scaling and precise measurement of the pipeline’s 3D model, enabling direct localization and quantification of defects within the sewer. The measurement results of three defects in the axial pipeline are shown in Figure 27. These defects, as well as parameters such as their locations, lengths, and widths, are visually depicted in the 3D model.

**Figure 28:**
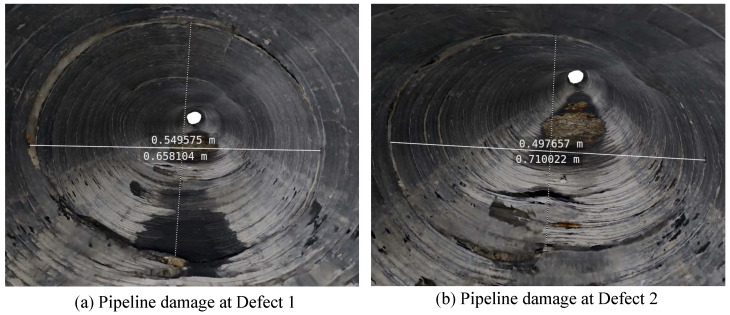
Three-dimensional measurement of internal pipeline defect damage. (**a**) Measurement of Defect 1. (**b**) Measurement of Defect 2.

Upon entering the interior of the 3D-modeled pipeline, a detailed assessment of the damage at Defects 1 and 2 was conducted. As shown in Figure 28, both defects exhibit cracks and deformation to varying degrees. Cracks can cause pipeline leakage or blockage, seriously affecting the normal operation of the pipeline. According to visual observations, Defect 1 is severe. The actual measurement results indicate that the damage in Defect 2 is relatively high and should be repaired first. Therefore, 3D measurement and evaluation are necessary to reduce errors associated with 2D visual judgment. Overall, the visualization function provided by 3D modeling facilitates a comprehensive quantitative analysis of pipeline defects and provides important guidance for subsequent excavation and repair operations.

## 5. Conclusions

To achieve fast and effective detection of drainage pipe defects in urban underground structure health monitoring and to comprehensively address the challenge of assessing complex urban pipeline conditions, this paper combines computer vision technologies such as deep learning and 3D geometric reconstruction to detect pipeline defects from 2D images and construct 3D pipeline scene models from multiview images. The proposed drainage pipe detection framework for the pipeline robot includes defect detection and deployment, reconstruction of the real 3D model of the pipeline, and measurement. The Sewer-YOLO-Slim algorithm is proposed for fast defect detection in complex urban drainage pipe environments. Through the improved YOLOv7-tiny model, combined with backbone network optimization, lightweight neck network design, and the introduction of the DyHead attention mechanism, the network was restructured, followed by pruning the improved model. These modifications resulted in a 60.2% reduction in model size, a 60.0% reduction in parameters, and a 65.9% reduction in FLOPs. Ultimately, the model size was reduced to only 4.9 MB, and the mAP value was increased by 1.5%, reaching 93.5%. The experimental results indicate that the improved algorithm achieves a smaller model size and faster detection speed while maintaining high accuracy, satisfying the requirements of practical engineering applications. Sewer-YOLO-Slim was deployed on edge devices and accelerated by TensorRT, achieving a real-time detection speed of 15.3 ms per image, which is highly important for practical engineering applications. Additionally, the integration of target detection techniques and 3D reconstruction strategies aids in assessing the condition of drainage pipes. The proposed bidirectional data acquisition 3D reconstruction approach significantly enhances the accuracy of the 3D model, with experiments showing a maximum measurement error of only 0.57 m. This research has made progress in utilizing visual sensors for pipeline defect detection and 3D modeling. However, several limitations remain, particularly in complex imaging environments, such as low-light conditions or water reflections within pipelines, which can compromise the imaging quality of visual sensors. These challenges lead to suboptimal defect detection and 3D modeling outcomes, reducing the accuracy and reliability of the detection system. Additionally, the 3D modeling process is computationally intensive and time-consuming, posing challenges for meeting real-time detection and rapid response requirements. Future research will focus on integrating information from multiple sensors, such as infrared and LiDAR, to address the limitations of visual sensors in challenging imaging conditions. To reduce resource consumption, leveraging 2D defect detection results for localized 3D modeling can significantly optimize computational efficiency. In terms of model generalization, incorporating more extensive training data and advanced optimization algorithms will enhance the accuracy and robustness of defect detection systems, providing a reliable foundation for ensuring the safe operation of urban underground pipelines.

## Figures and Tables

**Table 1:** The details of the datasets in the USDID.

Dataset	Defect Type					Number of Images
	Misplace	Obstacle	Root	Leaky	Fouling	
Training	3116	750	935	607	354	4466
Testing	1289	296	374	263	170	1902
Total	4405	1046	1309	870	524	6368

**Table 2:** Algorithm parameter settings.

Parameter	Value
Input resolution	416 × 416
Learning rate	0.001
Weight decay	0.0005
Epochs	300
Batch size	16
IoU	0.5

**Table 3:** Pruning experiment parameter settings.

Parameter	Value
Input resolution	416 × 416
Sparse learning rate	0.002
Sparse iterations	200
Channel pruning rate	0.5
Fine-tuning iterations	300

**Table 4:** Comparison of the impact of different lightweight backbone networks on YOLOv7-tiny.

Backbone	PrecisionL (%)	Recall (%)	mAP (%)	Model Size (MB)	Total Parameters (Million)	FLOPs (Giga)	Training Hours (h)
Original	92.1	84.6	92.0	12.3	6.03	13.2	9.847
GhostNet [56]	89.0	79.7	88.8	11.0	5.29	11.1	11.576
ResNet18 [57]	86.6	75.3	84.7	29.7	14.73	35.7	10.634
EfficientViT_M0 [58]	87.9	78.8	87.2	11.8	5.53	10.1	12.771
MobileNetV3 [59]	83.8	74.5	83.3	9.2	4.48	6.8	11.235
FasterNet-T0	93.5	85.2	93.4	11.6	5.66	11.6	6.278

**Table 5:** Different scheme designs.

Plan	FasterNet-T0	GSCConv + VoVGSCCP	DyHead
0	×	×	×
1	✓	×	×
2	✓	✓	×
3	✓	×	✓
4	✓	✓	✓

**Table 6:** Comparison of ablation experiments.

Model	Precision (%)	Recall (%)	mAP (%)	Model Size (MB)	Total Parameters (Million)	FLOPs (Giga)
Plan 0	92.1	84.6	92.0	12.3	6.03	13.2
Plan 1	93.5	85.2	93.4	11.6	5.66	11.6
Plan 2	93.3	85.1	93.1	8.1	3.89	7.7
Plan 3	94.3	87.3	94.2	11.5	5.61	11.3
Plan 4	93.9	87.7	93.8	10.2	4.94	9.0

**Table 7:** Comparative experimental results of different pruning rates.

Pruning Rate (%)	Precision (%)	Recall (%)	mAP (%)	Model Size (MB)	Total Parameters (Million)	FLOPs (Giga)
0	93.9	87.7	93.8	10.2	4.94	9.0
40%	93.8	87.2	93.7	6.6	3.14	5.9
50%	93.6	87.4	93.5	4.9	2.41	4.5
60%	90.5	82.5	89.4	3.8	1.86	3.0
70%	84.5	77.4	83.5	3.4	1.64	2.2
80%	53.9	51.0	51.9	3.2	1.52	1.8

**Table 8:** Comparison of mainstream detection algorithms.

Model	Precision (%)	Recall (%)	mAP (%)	Model Size (MB)	Total Parameters (Million)	FLOPs (Giga)
Faster-RCNN [15]	82.4	68.1	72.4	108.3	41.75	134.4
SSD [11]	91.7	75.2	87.1	92.6	24.15	116.2
YOLOv3 [60]	88.4	78.6	88.2	235.1	61.54	32.8
YOLOv4 [61]	87.8	75.4	86.1	244.5	63.95	59.9
YOLOv5l [62]	90.2	84.8	89.8	52.0	25.79	55.0
YOLOv7-tiny [61]	92.1	84.6	92.0	12.3	6.03	13.2
YOLOv7 [60]	95.3	86.8	94.4	74.8	36.5	103.2
YOLOv8n [63]	93.0	85.5	92.8	6.2	3.00	8.2
YOLOv9s [64]	93.6	86.6	93.4	15.2	7.17	26.7
YOLOv10n [65]	91.4	83.9	91.1	5.8	2.27	6.5
YOLOv11n [66]	90.7	83.4	90.5	5.5	2.58	6.3
Improved YOLO	93.9	87.7	93.8	10.2	4.94	9.0
Sewer-YOLO-Slim	93.6	87.4	93.5	4.9	2.41	4.5

**Table 9:** Comparison of different target detection devices.

Device	Use TensorRT	mAP	Speed
RTX 3090	No	93.5	22.5 ms
RTX 3090	Yes	92.7	14.0 ms
EA-B400	Yes	92.7	15.3 ms

**Table 10:** Verification of measurement results of one-way image 3D reconstruction model.

	Known		Verification	
	Inspection Well Diameter	Pipe Burial Depth	Pipe Diameter	Length of the Entire Pipe
Distance on 3D model (unit)	0.7622	2.1807	0.6034	20.1983
Reasoning distance (m)	-	2.06	0.57	19.08
Actual distance (m)	0.72	2.10	0.60	18.20
Error (m)	-	−0.04	−0.03	+0.88

**Table 11:** Verification of measurement results of two-way image 3D reconstruction model.

	Known		Verification	
	Inspection Well Diameter	Pipe Burial Depth	Pipe Diameter	Length of the Entire Pipe
Distance on 3D model (unit)	0.7601	2.2132	0.6175	18.6115
Reasoning distance (m)	-	2.10	0.58	17.63
Actual distance (m)	0.72	2.10	0.60	18.20
Error (m)	-	0	−0.02	−0.57

## Data Availability

Data will be made available upon reasonable request.

## Abbreviations

The following abbreviations are used in this manuscript:

FLOPs	Floating-Point Operations
mAP	mean Average Precision
VO	Visual Odometry
CCTV	Closed-Circuit Television
IMU	Inertial Measurement Unit
USDID	Urban Sewer Defect Image Database
BN	Batch Normalization
SIFT	Scale-Invariant Feature Transform
PnP	Perspective-n-Point
BA	Bundle Adjustment
SFM	Structure from Motion
MVS	Multiview Stereo
AP	Average Precision
TP	True Positive
FP	False Positive
FN	False Negative
